# Clinical practice, decision-making, and use of clinical decision support systems in invasive mechanical ventilation: a narrative review

**DOI:** 10.1016/j.bja.2024.03.011

**Published:** 2024-04-17

**Authors:** Mayur Murali, Melody Ni, Dan S. Karbing, Stephen E. Rees, Matthieu Komorowski, Dominic Marshall, Padmanabhan Ramnarayan, Brijesh V. Patel

**Affiliations:** 1 Division of Anaesthetics, Pain Medicine & Intensive Care, Department of Surgery & Cancer, Faculty of Medicine, Imperial College London, London, UK; 2 NIHR London In Vitro Diagnostics Cooperative, London, UK; 3 Respiratory and Critical Care Group, Department of Health Science and Technology, Aalborg University, Aalborg, Denmark; 4 Imperial Centre for Paediatrics and Child Health, London, UK; 5 Department of Anaesthesia & Critical Care, Royal Brompton Hospital, London, UK

**Keywords:** clinical decision support system, clinical practice, decision-making, implementation, invasive mechanical ventilation, personalised ventilation

## Abstract

Invasive mechanical ventilation is a key supportive therapy for patients on intensive care. There is increasing emphasis on personalised ventilation strategies. Clinical decision support systems (CDSS) have been developed to support this. We conducted a narrative review to assess evidence that could inform device implementation.

A search was conducted in MEDLINE (Ovid) and EMBASE. Twenty-nine studies met the inclusion criteria. Role allocation is well described, with interprofessional collaboration dependent on culture, nurse:patient ratio, the use of protocols, and perception of responsibility. There were no descriptions of process measures, quality metrics, or clinical workflow. Nurse-led weaning is well-described, with factors grouped by patient, nurse, and system. Physician-led weaning is heterogenous, guided by subjective and objective information, and ‘gestalt’. No studies explored decision-making with CDSS. Several explored facilitators and barriers to implementation, grouped by clinician (facilitators: confidence using CDSS, retaining decision-making ownership; barriers: undermining clinician's role, ambiguity moving off protocol), intervention (facilitators: user-friendly interface, ease of workflow integration, minimal training requirement; barriers: increased documentation time), and organisation (facilitators: system-level mandate; barriers: poor communication, inconsistent training, lack of technical support). One study described factors that support CDSS implementation.

There are gaps in our understanding of ventilation practice. A coordinated approach grounded in implementation science is required to support CDSS implementation. Future research should describe factors that guide clinical decision-making throughout mechanical ventilation, with and without CDSS, map clinical workflow, and devise implementation toolkits. Novel research design analogous to a learning organisation, that considers the commercial aspects of device design, is required.

**Editor's key points**

• Clinical decision support systems facilitate personalised treatment for patients receiving invasive mechanical ventilation. The authors sought evidence to guide their implementation.
• Role allocation and weaning practice are well described. Descriptions of process measures, quality metrics, or clinical workflow are absent. Facilitators and barriers to implementation are classified by clinician, intervention, and organisation.
• A coordinated approach grounded in implementation science is required. Future research should describe factors that guide clinical decision-making through all phases of ventilation, map clinical workflow, and consider commercial aspects of device design.

Invasive mechanical ventilation (IMV) is a key supportive therapy for patients,^1^ and is the main reason for patients requiring ongoing critical care admission.^2^^,^^3^ Mechanically ventilated patients represent a heterogenous group that differ in pathology, lung mechanics, and physiology,^4^^,^^5^ such that incorrect application of ventilatory strategies can cause morbidity and mortality.^6^ There is increasing emphasis on developing personalised ventilation strategies that are responsive to changes in patient state and are delivered in a repeatable way.^7^^,^^8^

The rationale for personalised ventilation management is supported in several domains. For instance, subgroup identification using clinical and plasma biomarker data, such as the hyper- and hypo-inflammatory subphenotypes in acute respiratory distress syndrome (ARDS),^9^ with the potential for therapeutic applications shown in several retrospective analyses.^10^^,^^11^ Physiological optimisation based on these disease states is effective; patients with diffuse, hyper-inflammatory ARDS are more likely to benefit from higher positive end expiratory pressure (PEEP),^12^ reducing dynamic strain and atelectotrauma,^13^ while those with focal, hypo-inflammatory ARDS benefit from lower PEEP, thus mitigating over-distension of the lung and haemodynamic changes from ventilation.^14^ In patients with the most severe ARDS, higher PEEP was associated with better survival,^15^ whereas ventilator strategy misaligned to lung morphology was associated with worse survival.^16^ In this context, optimising and personalising ventilator management by improving our understanding of disease state (such as ARDS subphenotypes) is key to improving patient management.

Several clinical decision support systems (CDSS) have been developed to help clinicians achieve this goal.^17^, ^18^, ^19^, ^20^ A CDSS is a computer system intended to augment decision-making by matching patient characteristics to a computerised clinical knowledge base; patient-specific recommendations are presented to the clinician.^21^ CDSS for mechanical ventilation take several forms, from standalone systems to an adjunct to the electronic health record, or a webpage that requires data input, with some ventilators also having built-in CDSS. They can support a variety of decisions, including titrating ventilator settings,^17^ supporting the implementation of lung-protective ventilation,^18^ assisting with weaning from mechanical ventilation,^19^ and those that support decisions in several areas.^20^ CDSS may optimise ventilator settings without clinician input (closed-loop),^22^^,^^23^ or may be used at the point of care, allowing clinicians to combine their knowledge with the suggestions provided by the CDSS (open-loop).^20^ Currently available commercial systems make use of varying methodology to offer clinical recommendations, including physiological models,^20^^,^^22^ rule-based systems,^23^^,^^24^ and neural networks.^25^^,^^26^ It is likely that machine learning methodology will become more prominent.^27^ In contrast to clinicians who have cognitive biases and limitations based on training and experience,^7^ CDSS can account for many factors to optimise care.

Because of their interaction with and need to involve clinicians in their decisions and the potential complexity this introduces to the clinical environment, this review will focus on open-loop CDSS. Because critical care units vary in size, resource, and staff composition, the use of open-loop CDSS requires an understanding of the best method of implementation. Methodology drawn from implementation science, the study of approaches promoting the systematic uptake of research findings and evidence-based practice,^28^ may be of benefit. It is distinct from, but shares characteristics with, quality improvement and dissemination methods.^29^ Various implementation models are described; process models include action models or ‘how-to-implement’ guides; determinant frameworks describe barriers and enablers that influence implementation outcomes; and several theoretical approaches also have also been published.^28^ Of relevance to CDSS is the technology acceptance model (TAM), which accounts for the end-user's behavioural intention to use a device (as a proxy for device acceptance), influenced by perceived usefulness of the device and ease of use.^30^^,^^31^ Subsequently updated to capture the impact of social norms (TAM2), a unified model, the unified theory of acceptance and use of technology (UTAUT) was devised.^30^ Such frameworks provide a useful construct to guide implementation efforts.

Successful implementation of open-loop CDSS requires identification of stakeholders and an understanding of current practice. This includes mapping clinical workflow; identifying factors that influence decision-making and who interacts with the ventilator; understanding the clinical environment; defining process measures to describe quality of decision-making; and defining quality metrics for benchmarking. Decisions which are not based on clinical information alone, but influenced by factors such as perceived accountability, staff mix, or stress, should be interrogated. This will lead to more intelligent, responsive CDSS design.

Thus, to inform the successful implementation of open-loop CDSS for mechanical ventilation, we conducted this narrative review to understand the following questions. (1) What factors influence clinical practice and decision-making in patients requiring invasive mechanical ventilation on intensive care? (2) What factors influence clinical practice and decision-making when using open-loop CDSS in patients requiring invasive mechanical ventilation on intensive care? (3) What are the facilitators and barriers to implementation for open-loop CDSS used for invasive mechanical ventilation?

## Search strategy and selection criteria

This study was considered a narrative rather than systematic review because of its broad scope, in contrast to systematic reviews which often seek to answer a single, specific question. Despite this, we applied the methodological rigour associated with a systematic review to ensure we extracted and reviewed most published evidence on this topic.

A search strategy was developed to identify studies that were published in MEDLINE (Ovid) and EMBASE (Supplementary Material). We included articles available in English, published up to 14 March 2023 in peer-reviewed journals. Studies were included if they (1) described clinical, technical, and human factors guiding decision-making during initiation, maintenance, and weaning of mechanical ventilation on intensive care, (2) described workflows for managing mechanical ventilation on intensive care, and (3) described facilitators or barriers to implementation, or implementation strategies for open-loop CDSS for mechanical ventilation.

Our main outcome was to describe factors that influence clinical practice and decision-making of clinicians who manage patients requiring mechanical ventilation, and to assess whether this changed with the introduction of CDSS. Our secondary outcome was to describe facilitators, barriers, and implementation strategies for the introduction of CDSS for mechanical ventilation.

Search results were saved to Rayyan (Qatar Computing Research Institute), a web-based application. Abstracts were independently screened for inclusion by one review author (MM). After exclusion of studies which did not meet the inclusion criteria and duplicates, full-text screening was carried out independently and in duplicate (by MM) and data were extracted into an Excel™ (Microsoft Corp., Redmond, WA, USA) spreadsheet.

## Data analysis

In addition to general study characteristics, data were collected on study objectives, methodological approach, and the type of intensive care unit and staff group being assessed. To assess our main outcome, we extracted data on the factors that guide decision-making during mechanical ventilation and the use of CDSS. We also documented descriptions of clinical workflow, the approach to CDSS implementation, and any reported barriers and facilitators. All studies which reported any data that answered either our primary or secondary outcomes were included in the final analysis.

## Characteristics of included studies

**Fig 1:**
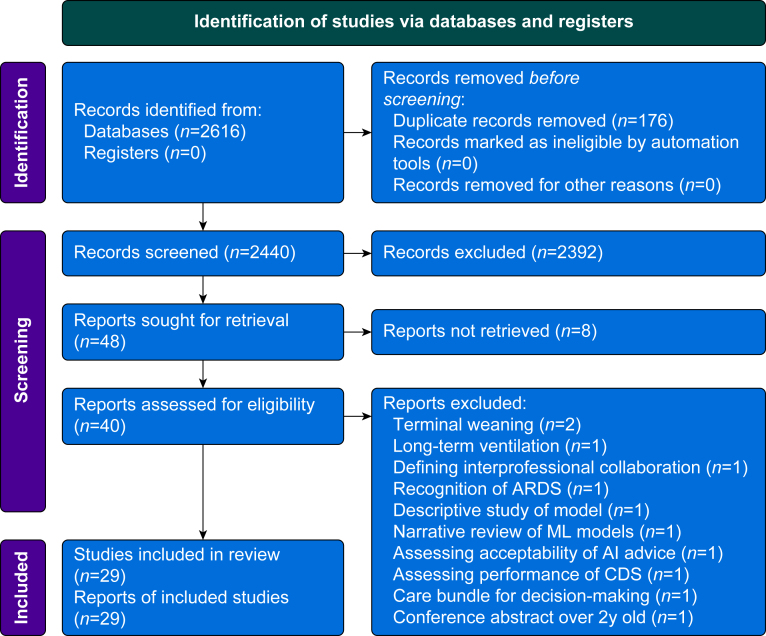
Flow chart of study selection. AI, artificial intelligence; ARDS, acute respiratory distress syndrome; CDSS, clinical decision support system; ML, machine learning.

A total of 2616 studies were identified through the database search, and 2390 studies were excluded during the abstract screening phase. After removal of duplicates, those that did not meet the inclusion criteria, and those for which we could not retrieve any records, we reviewed 40 studies for full-text screening, after which a further 11 studies were excluded (Fig. 1) and a total of 29 were included (see Table 1 for a list of identified articles and their characteristics). The most common reason for exclusion at full-text review was because the studies assessed terminal weaning, outside of the intensive care setting. Of the included studies, eight described role allocation for mechanical ventilation; 27 described factors guiding decision-making during mechanical ventilation; four described the facilitators and barriers for the implementation of new interventions, including CDSS, in critical care; no studies described mechanical ventilation workflow, and one described implementation strategies for CDSS (although two further studies described implementation strategies for new care processes). Eight studies assessed the nurse staff group, four assessed physician decision-making, and 15 assessed more than one staff group. The most assessed area was weaning of mechanical ventilation (18 studies), and the most common data collection methods were survey (12 studies) and interview (10 studies).

**Table 1:** Identified articles and their characteristics. CDSS, Clinical decision support systems.

No.	Title	Authors	Year	Journal	Study design	Staff group	Topic assessed
**1**	Feasibility of implementing Extubation Advisor, a clinical decision support tool to improve extubation decision-making in the ICU: a mixed-methods observational study	Sarti AJ, Zheng K, Henry CL and colleagues	2021	*BMJ Open*	Mixed-methods: observational, survey and interviews	Respiratory therapists	Feasibility of CDSS
**2**	ABCDE and ABCDEF care bundles: a systematic review of the implementation process in intensive care units	Moraes F, Marengo LL, Moura MDG and colleagues	2022	*Medicine*	Systematic review	N/A	Implementation of care bundles, facilitators and barriers to implementation, implementation strategies
**3**	Mechanical ventilation, weaning practices, and decision making in European PICUs	Tume LN, Kneyber MCJ, Blackwood B and colleagues	2017	*Paediatric Critical Care Medicine*	Survey	Multi-professional	Role allocation, decision-making
**4**	Continuous care and patients' basic needs during weaning from mechanical ventilation: a qualitative study	Khalafi A, Elahi N, Ahmadi F	2016	*Intensive & Critical Care Nursing*	Semi-structured interviews	Multi-professional	Weaning
**5**	Understanding nurses' decision-making when managing weaning from mechanical ventilation: a study of novice and experienced critical care nurses in Scotland and Greece	Kydonaki K, Huby G, Tocher J, Aitken LM	2016	*Journal of Clinical Nursing*	Descriptive ethnographic study	Nurses	Weaning
**6**	Weaning from mechanical ventilation: factors that influence intensive care nurses' decision-making	Tingsvik C, Johansson K, Martensson J	2015	*Nursing in Critical Care*	Semi-structured interviews	Nurses	Weaning
**7**	Weaning from mechanical ventilation: a scoping review of qualitative studies	Rose L, Dainty KN, Jordan J, Blackwood B	2014	*American Journal of Critical Care*	Scoping review	Multi-professional	Weaning
**8**	Critical care nurses management of prolonged weaning: an interview study	Cederwall CJ, Plos CK, Rose L and colleagues	2014	*Nursing in Critical Care*	Semi-structured interviews	Nurses	Weaning
**9**	Perceived decisional responsibility for mechanical ventilation and weaning: a Norwegian survey	Haugdahl HS, Storli S, Rose L and colleagues	2014	*Nursing in Critical Care*	Survey	Multi-professional	Role allocation, weaning
**10**	Role responsibilities in mechanical ventilation and weaning in pediatric intensive care units: a national survey	Blackwood B, Junk C, Lyons JDM and colleagues	2013	*American Journal of Critical Care*	Survey	Multi-professional	Role allocation, weaning
**11**	Anaesthetists' perceptions of facilitative weaning strategies from mechanical ventilator in the intensive care unit (ICU): a qualitative interview study	Pettersson S, Melaniuk-Bose M, Edell-Gustafsson U	2012	*Intensive Care & Critical Care Nursing*	Interview	Physician	Weaning
**12**	Decisional responsibility for mechanical ventilation and weaning: an international survey	Rose L, Blackwood B, Egerod I and colleagues	2011	*Critical Care*	Survey	Multi-professional	Role allocation, weaning
**13**	The factors which influence nurses when weaning patients from mechanical ventilation: findings from a qualitative study	Lavelle C, Dowling M	2011	*Intensive & Critical Care Nursing*	Semi-structured interviews	Nurses	Weaning
**14**	Accuracy and reliability of extubation decisions by intensivists	Tulaimat A, Mokhlesi B	2011	*Respiratory Care*	Survey	Physicians	Weaning, extubation
**15**	Workforce profile, organisation structure, and role responsibility for ventilation and weaning practices in Australia and New Zealand intensive care units	Rose L, Nelson S, Johnston L and colleagues	2008	*Journal of Clinical Nursing*	Survey	Nurses	Decision-making, role allocation, weaning
**16**	A comparative study examining the decision-making processes of medical and nursing staff in weaning patients from mechanical ventilation	Taylor F	2006	*Intensive & Critical Care Nursing*	Semi-structured interviews	Multi-professional	Weaning
**17**	The decision-making processes of nurses when extubating patients following cardiac surgery: an ethnographic study	Hancock HC, Easen PR	2006	*International Journal of Nursing Studies*	Ethnographic study	Nurses	Weaning, extubation
**18**	A study exploring factors which influence the decision to commence nurse-led weaning	Gelsthorpe C, Crocker C	2004	*Nursing in Critical Care*	Interviews	Nurses	Weaning
**19**	Protocolized weaning from mechanical ventilation: ICU physicians' views	Blackwood B, Wilson-Barnett J, Trinder J	2004	*Journal of Advanced Nursing*	Interviews	Physicians	Weaning
**20**	Evaluation of the perceived barriers and facilitators to timely extubation of critically ill adults: an interprofessional survey	Balas MC, Tate J, Tan A and colleagues	2021	*Worldviews on Evidence-Based Nursing*	Survey	Multi-professional	Extubation, role allocation
**21**	Multi-factorial barriers and facilitators to high adherence to lung-protective ventilation using a computerized protocol: a mixed methods study.	Knighton AJ, Kean J, Wolfe D and colleagues	2020	*Implementation Science Communications*	Interviews	Multi-professional	Lung-protective ventilation; facilitators and barriers to implementation
**22**	Perceived responsibility for mechanical ventilation and weaning decisions in intensive care units in the Kingdom of Saudi Arabia	Alkhathami M, Al-Haddad M, Alenazi A	2022	*Chest*	Survey	Multi-professional	Role allocation, weaning
**23**	Understanding interprofessional decision-making processes and perceptions of oxygenation for acute respiratory failure	Curtis B, Rak K, Richardson A and colleagues	2020	*America Journal of Respiratory and Critical Care Medicine*	Semi-structured interviews	Multi-professional	Role allocation
**24**	Barriers and facilitators to timely extubation of critically ill adults	Tate J, Balas M, Tan A and colleagues	2020	*America Journal of Respiratory and Critical Care Medicine*	Survey	Multi-professional	Extubation
**25**	Patient involvement in micro-decisions in intensive care	Karlsen MMW, Happ MB, Finset A and colleagues	2020	*Patient Education & Counselling*	Observation of video recordings	Multi-professional	Decision-making
**26**	Nurses' near-decision-making process of postoperative patients' cardiosurgical weaning and extubation in an Italian environment	Villa G, Manara D, Palese A	2012	*Intensive and Critical Care Nursing*	Descriptive ethnographic	Nurses	Role allocation, weaning, extubation
**27**	Clinical decision-making and mechanical ventilation in patients with respiratory failure due to an exacerbation of COPD	Perrin F, Renshaw M, Turton C	2003	*Clinical Medicine*	Survey	Physicians	Initiation of ventilation
**28**	Factors influencing the patient during weaning from mechanical ventilation: A national survey	Mårtensson IE, Fridlund B	2002	*Intensive and Critical Care Nursing*	Survey	Multi-professional	Weaning
29	Developing and implementing computerized protocols for standardization of clinical decisions	Morris AH, Hudson B	2000	*Annals of Internal Medicine*	Descriptive ethnographic	Multi-professional	Facilitators and barriers to implementation

## Factors guiding decision-making and role allocation during mechanical ventilation

The factors that guide decision-making and role allocation during mechanical ventilation are summarised in Supplementary Table S1, but can be broadly divided into decision-making and the decision-makers.

## Decision-making

Decision-making across adult and paediatric intensive care is relatively well described and shows similarities across the literature. Eight key ventilation decisions are defined, and responsibility for these decisions is generally spread. Although senior physicians are most likely to select initial ventilator settings and weaning method, and lead decisions about extubation readiness or the initiation of noninvasive ventilation,^32^^,^^33^ nurses lead or are more likely to collaborate with physicians when determining weaning readiness and failure, or titrating ventilator settings.^32^, ^33^, ^34^, ^35^ Nursing autonomy varies, but is highest in units where the nurse:patient ratio is 1:1.^32^^,^^33^ When adjusting the ventilator, nurses most commonly adjusted the fraction of inspired oxygen (FiO_2_) and ventilatory frequency,^32^^,^^35^ although nurses in Australia and New Zealand felt more empowered to adjust pressure support compared with their European colleagues.^35^ However, the perceived role in decision-making varies by professional group. A Norwegian study found nursing directors perceived their autonomy and influence to be higher than that perceived by physicians.^36^

Type of decision also varies according to professional group. When assessing understanding of hyperoxaemia in critically unwell patients, Curtis and colleagues^37^ found nurses assessed patients through objective and subjective criteria and were unlikely to operate the ventilator; respiratory therapists followed protocol while implementing goals of care; physicians implemented condition-specific treatment that did not rely on protocolised management.

## Decision-makers

**Fig 2:**
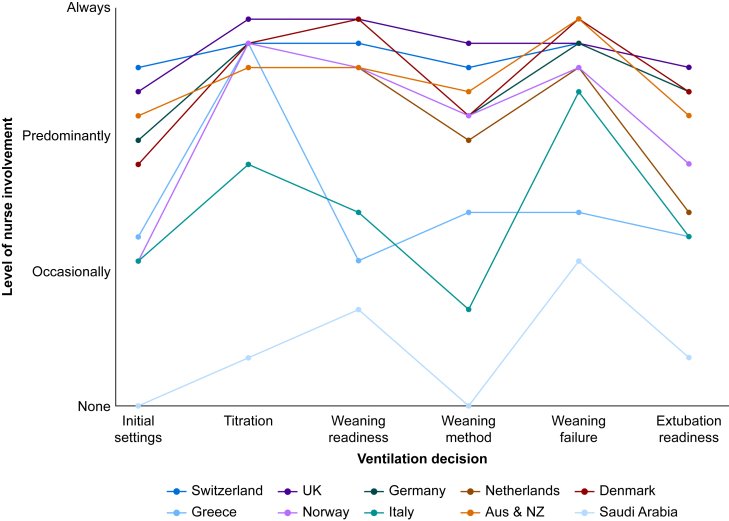
Schematic representation of nurse involvement in invasive mechanical ventilation decision-making by country in adult patients. This summarises the findings of the literature identified in Supplementary Table S1 by country. It illustrates that nurses are more likely to be involved with titration of ventilator settings, assessing weaning readiness, and weaning failure. Nurses in Switzerland and the UK have the greatest level of invasive mechanical ventilation (IMV) decision-making involvement, whereas Saudi Arabia, Greece and Italy have the lowest.

Country influenced the decision-making profile, with Switzerland and the UK most likely to be collaborative, and Italy and Norway most likely to be physician-led.^34^ The ethnographic study by Villa and colleagues^38^ explored the role of nurses in Italy, and found nurses rarely took the initiative to wean or extubate; instead offering recommendations that were authorised by physicians. Alkhathami and colleagues^39^ assessed perceptions of roles and responsibilities in Saudi Arabia; in contrast to Western Europe, they found physicians and respiratory therapists collaborated on key decisions, with limited nursing involvement. The differences in nurse influence on mechanical ventilation decision-making by country is illustrated in Figure 2.

The evidence demonstrates that culture is key, highlighted by a survey by Balas and colleagues^40^ exploring reasons for delay to extubation in the USA: decreasing clinician perception of reprimand or condemnation for failed extubation was considered important in encouraging clinical decision-making and reducing delays.

## Factors that guide nurse-led weaning of mechanical ventilation

**Fig 3:**
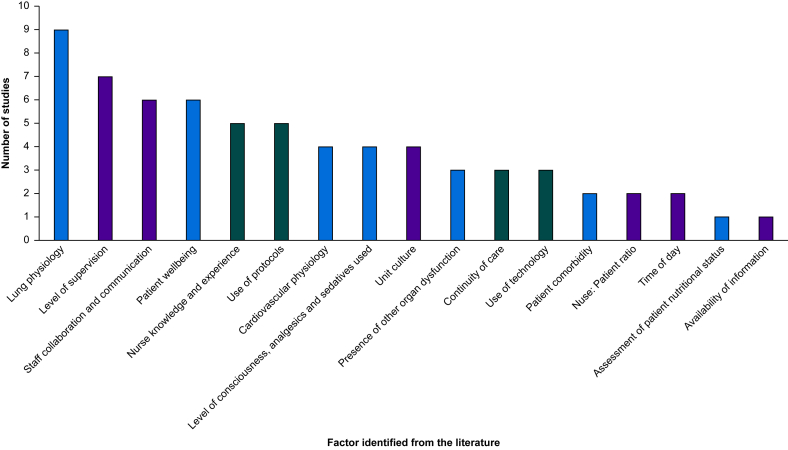
Factors influencing nurse-led weaning from invasive mechanical ventilation. Factors influencing nurse-led weaning can be grouped into those affecting the patient (blue bars), nurse (dark green bars), and system (purple bars).

Factors guiding nurse-led weaning from invasive mechanical ventilation are summarised in Figure 3.

Nurse-led weaning is guided by physiological criteria that identify whether a patient is ‘weanable’, including gas exchange, work of breathing, lung condition, cardiovascular stability, and signs of infection.^36^^,^^41^^,^^42^ Subjective knowledge of the patient, arrived at through regular assessment and clinical examination,^43^ and objective data, from observations and investigations, are combined.^44^ Other patient factors that guide weaning include an understanding of the patient's medical history,^45^ nutrition, metabolism, measurable parameters, and psychological well-being,^46^ and an assessment of the likelihood of clinical deterioration.^36^ Not all nurses use each piece of evidence, less experienced nurses require more cues, and criteria were discerning at different stages of weaning^41^ (see Supplementary Table S2).

In addition to these criteria, heterogeneity in nursing practice, influenced by professional knowledge, level of training, role and scope of practice, and experience, personality, and confidence^44^^,^^45^^,^^47^ play a role. Nursing practice is in turn influenced by time spent with the patient, key to understanding and responding to their condition^48^; recognising fixed criteria from protocols which cannot always be applied because of variation in patient presentation, and seeking an individualised approach instead^43^^,^^44^^,^^48^, ^49^; and maintaining quality of care through shift changes and handover.^48^ Weaning decisions are often arrived at through a trial-and-error approach.^41^ Two psychological concepts describe differences in approach when adjusting the ventilator: focus gambling (changing more than one setting at once) and conservative focusing (changing a single setting).^41^ Professional accountability, the ability to justify decision-making, particularly when working off protocol, and taking overall responsibility for weaning, also determine a nurse's scope of practice and their degree of independent decision-making.^50^

System or structural issues, including the conditions or environment under which the clinical team works, interprofessional communication and collaboration (particularly with physicians), resource availability, and the use of and compliance with unit-based protocols to drive clinical practice, also play a role^42^, ^43^, ^44^, ^45^^,^^47^(Supplementary Table S2).

## Factors that guide physician-led weaning of mechanical ventilation

Physicians use three types of information to arrive at weaning decisions: empirical objective (physiological measurements), empirical subjective (clinical examination), and abstract (intuition derived from experience).^44^^,^^51^ Physicians aim to offer care that is flexible and individually tailored to each patient.^52^ Facilitative weaning strategies are arrived at through several approaches; the instrumental (through optimisation of physiology), interacting (through the physician's social interactions), process-orientated (goal-related), and the structural (individual and organisational competence) (see Supplementary Table S2).^52^ Physicians also use the experience of members of the multidisciplinary team to relay information and arrive at decisions.^44^

However, this individualised approach creates heterogeneity. Tulaimat and Mokhlesi^53^ used clinical vignettes to assess whether clinicians would extubate; agreement between any two clinicians was fair and was highest between clinicians from the same institution. A third relied on the breathing pattern whilst the patient was on pressure support, half relied on the acid–base status, whereas fewer relied on mental state assessment or volume of secretions. Apart from revealing significant heterogeneity in the factors required to guide extubation, accuracy of physician decisions to extubate was low^53^ (Supplementary Table S2).

## Workflow

We did not identify any studies that described the workflow for mechanical ventilation. However, a study by Tate and colleagues^54^ described factors leading to delayed extubation; because most spontaneous breathing trials (SBTs) took place overnight, there were significant delays between the SBT and the decision to extubate (average time 3–6 h). Most clinicians felt this was too long and contributed to poor patient outcome. Causes for the delays involved system issues: for example, deferring the decision to the day team, or waiting for a senior clinician to be present.^54^

## Facilitators and barriers to implementation of clinical decision support systems

Few CDSS are used for the management of ventilation,^33^ though automated weaning modes were used in more than half of surveyed European critical care units.^34^ The facilitators and barriers to implementation are summarised in Table 2.

**Table 2:** Facilitators and barriers to implementation of CDSS. CDSS, Clinical decision support systems.

	Facilitators	Barriers
**Clinician**	• Manageable workload with CDSS • Feeling of ownership and responsibility over clinical management • Underlying knowledge • Ability to detect condition requiring application of CDSS • Confidence and intent to use tool • Scholarly commitment to research • Incentivisation for enrolling patients • Prestige derived from association with research • Acceptance of the need to standardise clinical processes in order to reduce costs • Perceived educational value of rigorous clinical process monitoring	• Concerns over job security • Discomfort with perceived judgement (undermines complexity of role in extubation decision-making, caution over using standardised tool that does not treat each patient individually) • Uncertainty over the use of CDSS and their purpose • Uncertainty over when appropriate to depart from recommendations • Variation in practice between autonomous practitioners • Lack of appreciation of the limitations of human decision-making • Disproportionate impact of personal (anecdotal) experiences • Tendency to focus on infrequent but possible clinical scenarios that are not accommodated by protocol • Hubris among clinicians defending their autonomy • Medical culture that relies on the expert method of decision-making
**Intervention**	• Previous favourable patient outcomes • Ease of use and ease of data entry process (user-friendly interface) • Workflow integration (data entry ∼10 m) • Data completeness (model considers factors that are relevant to extubation readiness that are not routinely considered) • Ease of use with minimal technical training • Perceived to be advantageous to patients • Perceived to improve clinician self-efficacy and confidence	• Mixed acceptability of predictive model • Excessive documentation/workload associated with intervention • Perceived risks to patient safety • Not perceived to respond quickly or efficiently to patient state • Unclear practical application • Resentment at adopting a care process clinician did not assist in developing • Failure to account for all important possibilities in the clinical situation
**Organisation**	• Leadership/senior clinician involvement and support—perception that use of CDSS is a system-level, top-down mandate • Peer pressure because of changing standards of care • Team feels safe and supported • Training and practice-oriented training • Strengthening organisational culture • Performance evaluation • Continuing education • Regular audit • Dedicated implementation team	• Communication gap over implementation—centralised, poor flow, rumours, and misconceptions over study purpose • Training gaps and high staff turnover: varying work schedules means not everyone receives training • Lack of planning and support by senior leadership, with local clinical leaders having limited accountability • Team members not involved until the end of implementation cycle • Lack of dedicated implementation team • Not supported by organisational culture and values • Resentment from clinicians over loss of autonomy • Lack of clarity at organisation level about definition of success using CDSS • Peer pressure—local opponents having a meaningful impact on attitudes using CDSS • Perception that additional data (e.g. laboratory tests) required to use CDSS • Lack of transparency • Insufficient technological infrastructure • Lack of technical support

Two of the systems identified in the literature were Extubation Advisor, a web-based CDSS,^55^ and a computerised lung-protective ventilation clinical decision support tool for patients with ARDS.^56^ Despite differences in design, functionality, and the types of decisions supported, facilitators and barriers to implementation can be broadly classified into those that influence the clinician, the intervention itself, and the organisation. Clinician facilitators included confidence using the CDSS, understanding the underlying disease pathology, and retaining ownership over decision-making, whereas barriers included a perceived undermining of the clinician's role, concerns over job security, and uncertainty over when to move off protocol. Facilitators for the intervention itself included a user-friendly interface, ease of workflow integration, and efficiency and ease of use with minimal training, whereas barriers included increased time required for documentation. System facilitators included the perception that implementation of the CDSS was a system-level mandate, whereas barriers included poor communication from clinical leads, inconsistency in training received because of varying work schedules, local leaders having limited accountability, and limited availability of technical support.^55^^,^^56^

Similarities were noted in the description by Morris^57^ of the barriers and facilitators to the implementation of computerised protocols in critical care. Clinician facilitators included a personal commitment to research and quality improvement; intervention facilitators included evidence of good outcomes with the device; and system facilitators included incentivisation for enrolling patients, the influence of senior clinicians, and the role of peer pressure. Clinician barriers included the disproportionate weight applied to personal experience and pride, or hubris, among clinicians; intervention barriers included the exclusion of end-users from device development and excessive complexity and data entry requirements; and system barriers included insufficient technological infrastructure.^57^

A systematic review assessing the implementation of care bundles identified several factors which can be applied to CDSS implementation.^58^ Facilitators to implementation focused largely on system factors, including the support of senior leadership; multidisciplinary, practice-orientated training; regular performance evaluation; continuing education; regular audit; a dedicated implementation team; and a facilitative unit culture. Fear of harm to patients was identified as a key barrier to the implementation of any new care process.

## Implementation strategies for clinical decision support systems

Morris^57^ cites several factors that support implementation of CDSS. These include explicit logic, rules, and thresholds for variables that avoid the need for clinical judgement or opinion; inclusion of all outcomes for which protocol rules apply; generation of explicit, unambiguous instructions; and point-of-care application that is integrated with hospital electronic systems.^57^ The importance of identifying, capturing, and reviewing clinician non-adherence to CDSS is discussed as part of a reliable monitoring and evaluation programme.^57^ This information, along with data on protocol performance, provides evidence for iterative improvement. He also emphasises the importance of technical and clinical support availability, at any time.^57^

Two papers suggest implementation approaches for care bundles and processes in the intensive care unit, which might be applied to CDSS. Moraes and colleagues^58^ suggest regular educational meetings, supported by materials such as posters, with continuing education provided through case studies and applications in practice. Other interventions include involvement of local leadership, use of audit, expert review, patient-oriented interventions, and customised strategies for each department.

## Discussion

Our narrative review of 30 studies has revealed several key findings, while highlighting gaps in our understanding. Mechanical ventilation is a complex clinical intervention, with multiple decision nodes, operated by staff from different professional backgrounds with a range of knowledge and experience. Significant heterogeneity exists throughout the process, including decisional responsibility, information used to arrive at decisions, risk and decision thresholds. CDSS for mechanical ventilation are uncommon and face multiple barriers to implementation.

Our review of the literature has shown that role allocation for ventilation management is relatively well described. Nurses are most likely to lead decisions around weaning readiness and failure; and physicians are most likely to lead decisions on initial ventilator settings, weaning method, and extubation readiness. Interprofessional collaborative decision-making is important, and is similar between adult and paediatric critical care, but varies between countries, with western European nations encouraging greater nursing autonomy (Fig. 2). Differences in training requirements and cultural factors (e.g. more hierarchical or paternalistic models of healthcare) might explain this diversity of findings. Nursing autonomy and influence on decisions are dependent on nurse:patient ratio and are more likely to be present where the ratio is 1:1. Unit culture, and reducing perception of reprimand, leads to greater autonomy by junior staff, and suggests differences could be found between units of varying leadership models, size, staff experience, and composition; this represents an area of future research. There was no information on clinical workflow, although one study explored reasons for delay in time to extubate; and no studies defined process measures of ventilation, outside of morbidity, mortality, and weaning success (which itself has several definitions).

Weaning of mechanical ventilation has been explored in more detail, in particular, factors that influenced nurse-led weaning and are broadly grouped into patient, nurse, and system factors (Fig. 3). In contrast, exploration of physician decision-making around weaning was limited, with available studies showing significant heterogeneity in practice, description of tailored strategies, and no evidence exploring the impact of clinician specialty, experience, or level of training. Physicians, although using a combination of objective and subjective information, appeared more likely to draw on intuition derived from patient factors and their own experience. Our review suggests weaning of mechanical ventilation is where the value of CDSS may lie. Early liberation from the ventilator reduces the risk of nosocomial pneumonia and ventilator-induced lung injury,^19^ and costs associated with prolonged stay on intensive care.^19^^,^^59^ However, premature discontinuation of ventilation can lead to respiratory muscle fatigue, poor gas exchange, and increased morbidity and mortality.^60^^,^^61^ The success rate of physician-led weaning is low, and reported to be <50% in some studies^62^; thus, CDSS may prioritise this aspect of mechanical ventilation.

The difference in physician practice is demonstrated by a study which used clinical vignettes to assess whether clinicians would administer fluid or vasopressors in septic shock.^63^ The authors found there was extreme variability between the combination of quantitative metrics used. Physicians requested data which they subsequently said they would not use. More than one-third of respondents relied on ‘gestalt’ to make clinical decisions. The decision to start vasopressors was often dependent on systems considerations, including patient location, availability of support staff, and access to technology. Fluid administration varied hugely, in terms of the initial bolus, total volume administered, and the volume after which vasopressors were started.^63^ By exploring clinical decision-making, which is, in some respects, binary (i.e. give either fluid or vasopressor), and revealing huge variation in practice, this illustrates the complexity of attempting to describe clinical behaviour. This is even more challenging for mechanical ventilation, which has many variables to consider.

Allerød and colleagues^64^ evaluated clinicians' opinions towards ventilator settings generated by a decision support system or their colleagues from 10 simulated cases. As the vignettes were the same, and the CDSS physiological models behaved identically, the study attempted to factor out variability in patient state and focus on decision-making alone. They found clinician preferences towards ventilator settings and the resulting simulated values were significantly different. Clinicians had a poor opinion of both the advice provided by the CDSS and their colleagues, with advice considered unacceptable in a third of cases.^64^ Clinicians were also asked to rank each piece of advice, with heterogeneity found in the rankings, confirming clinicians rarely agree.^64^

The picture is further complicated by the variability in definitions within critical care and those for treatment criteria. A systematic review by Hakim and colleagues^65^ found significant heterogeneity in the definition of acute respiratory failure and the criteria for intubation, with different physiological measurements and descriptions of increased respiratory effort used across 50 trials, and variable criteria used to judge the need for intubation. Standardising criteria that govern when to intervene will be key to reducing heterogeneity in practice.

Although no studies explored decision-making while using CDSS, several explored facilitators and barriers to the implementation of CDSS and care processes in critical care. These can be broadly categorised into those at the level of the clinician, the intervention, and the organisation. Although we found studies that illustrated the clinical and economic utility of CDSS,^19^^,^^59^ no studies described possible workflows for CDSS. Although outside the scope of this review, Arnal and colleagues described how INTELLiVENT-ASV, a closed loop CDSS, significantly reduced the number of manual ventilator setting changes,^66^ illustrating the potential for such devices to augment clinical workflow.

One study described factors to support implementation of open-loop CDSS; these included integration with existing electronic health records, robust monitoring and evaluation, adequate technical and clinical support, and regular interrogation of clinician interaction with the CDSS, including non-adherence.^57^ Clinician feedback to improve CDSS can be described using the concept of the ‘learning organisation’; this describes how organisations collaborate with employees to drive collectively accountable change, and consists of disciplines including team learning and systems thinking.^67^ It is important to consider how this approach fits within a modern randomised controlled trial (RCT), as it necessarily breaks blinding and independence rules. Novel approaches to evidence generation and implementation, therefore, are required. We found no studies that used implementation strategies or frameworks, such as the UTAUT.

Despite the increasing availability of evidence for interventions which are shown to be cost effective, many fail to be implemented into practice.^68^ For clinical interventions, uptake and acceptance is challenging and slow. Key to overcoming these barriers is demonstrating clinical effectiveness for the patient and economic value for the healthcare organisation. To reduce ‘second translational gap’ (the disconnect between development and use in clinical practice), an approach that makes use of implementation science should be considered. Such an approach should be multidisciplinary, to identify factors that impact the patient, clinician, organisation, the broader healthcare community, and policy environment.^29^ We postulate that such an approach will improve the uptake for CDSS for mechanical ventilation and enable adoption of best practice and shared learning.

This review also recognises the challenge of addressing this issue without considering commercial aspects. Decision-aiding devices are classified as medical devices and cannot undergo trials unless they receive adequate regulatory approval. For example, in the UK, medical devices may require a clinical investigation (a prospective clinical trial) to prove safety and performance under normal conditions, within its intended scope of use. A clinical investigation must have approval from the Medicines and Healthcare products Regulatory Agency (MHRA) and Health Research Authority (HRA) before it can be used, including an independent ethical opinion from a research ethics committee. Apart from the time required for these approvals to be granted, there is a not-insubstantial fee associated with the application. Such approval is expensive, outside the funding remit and expertise of scientific employees. Consequently, scientific progress through trials is only possible with commercial collaboration, which is dependent on benefits for commercial partners in terms of the business case, and the necessary intellectual property protection to allow for competitive advantage. This can present a barrier to progress, particularly if benefits to patient care or cost saving are not immediately apparent, or if many years of investment are required before return on investment is seen. A new culture of exploration is required for decision tools where tight regulation is compensated for by regular review of cases. This would match with a learning organisation rather than an RCT-type trial and align more closely with ‘usual’ clinical practice.

This narrative review had several strengths; it used a robust search strategy to examine two biomedical databases, reviewing >2500 studies, and it had clear objectives, which were addressed from the gathered evidence. Limitations include reproducibility of our findings and applicability outside the setting of high-income healthcare settings.

In conclusion, this narrative review has highlighted gaps in our understanding of ventilation practice and the need for a coordinated, multilevel approach grounded in implementation science to support the use of open-loop CDSS. Future research should describe the factors that guide clinical decision-making at all stages of mechanical ventilation, both with and without CDSS, map clinical workflow, and devise implementation toolkits to guide use in clinical practice. Novel research design, analogous to a learning organisation, that consider the commercial aspects of device design, is required.

## Authors’ contributions

Conception and design of the work: MM, MN, PR, BP

Acquisition of the data: MM

Analysis and interpretation of the data: MM, DSK, SER, MK, DM

Drafted the final manuscript: MM

Substantively revised the final manuscript: all authors

All authors have approved the submitted version and have agreed both to be personally accountable for the author's own contributions and to ensure that questions related to the accuracy or integrity of any part of the work, even ones in which the author was not personally involved, are appropriately investigated, resolved, and the resolution documented in the literature.

## Acknowledgements

We would like to thank Nia Roberts, Bodleian Health Care Libraries, for her advice on databases and invaluable help in putting together the search strategy for this review.

## Declaration of interest

MK consults for Philips Healthcare, and receives honoraria from GE Healthcare.

## Data availability

All data generated or analysed during this study are included in this published article and its supplementary information files.

## Funding

The National Institute for Health & Care Research Medtech and InVitro diagnostics Co-operative (MICs) programme (RDB21 79560, 01 Jan 2018–31 Mar 2024). The views expressed are those of the author(s) and not necessarily those of the NHS, the National Institute for Health and Care Research (NIHR), or the Department of Health.
