# Supplementary material for: Clinical practice, decision-making, and use of clinical decision support systems in invasive mechanical ventilation: a narrative review
Source: Br J Anaesth. 2024 Apr 17;133(1):164–77. doi: 10.1016/j.bja.2024.03.011 (PMC11213991; doi:10.1016/j.bja.2024.03.011)
Supplement: Multimedia component 1 [file mmc1.docx]

**Supplementary Table 1 – Role allocation during mechanical ventilation**

| Title | Authors | Year | Factors identified |
| --- | --- | --- | --- |
| Mechanical Ventilation, Weaning Practices, and Decision Making in European PICUs | Tume L, Kneyber MCJ, Blackwood B et al | 2017 | 8 key ventilation decisions:   - Determining initial ventilator settings - Titration of ventilator settings - Weaning readiness+ - Weaning method* - Weaning failure+ - Extubation readiness* - Initiation of NIV* - Weaning/ discontinuation of NIV+   * most likely to be led by physicians  + most likely to be led by nurses  Ventilator management:   - Nurses most likely to adjust FiO_2_ and respiratory rate - Highest autonomy when nurse:patient ratios 1:1 |
| Perceived decisional responsibility for mechanical ventilation and weaning: a Norwegian survey | Haugdahl HS, Storli S, Rose L et al | 2014 | Nurses perceive the autonomy and influence of nurses’ ventilator decisions higher than physicians.  Respondents agreed nurses collaborate with physicians during:   - Assessment of patient response to ventilator changes - Titration of ventilator settings - Recognising weaning failure   Physician directors perceive less collaborative decision-making on:   - Weaning method - Weaning readiness - Readiness to extubate   Both groups acknowledge factors for determining weaning tolerance include:   - Knowing the patient - Assessment of work of breathing - Patient well-being - Observation of clinical deterioration |
| Role responsibilities in mechanical ventilation and weaning in paediatric intensive care units: a national survey | Blackwood B, Junk C, Lyons J et al | 2013 | Nurses collaborated with physicians on:   - Adjustment of ventilator settings - Determination of weaning readiness - Determination of weaning failure   Competency training required in 35% of PICUs before nurses can adjust ventilator |
| Decisional responsibility for mechanical ventilation and weaning: an international survey | Rose L, Blackwood B, Egerod I et al | 2011 | interprofessional collaboration between nurses and physicians most common approach to:   - Initial ventilator settings - Titration of ventilator settings - Determination of extubation readiness - Weaning method - Recognition of weaning failure   Collaboration increased if weaning protocols used.  Collaboration highest when nurse:patient ration 1:1  Country influenced nurse autonomy: Switzerland and UK most autonomous, Italy and Norway least |
| Workforce profile, organisation structure and role responsibility for ventilation and weaning practices in Australia and New Zealand intensive care units | Rose L, Nelson S, Johnston L | 2008 | Inter-professional collaborative decision-making predominant for key ventilator decisions, including assessment of weaning extubation readiness.  Nurses describe high level of autonomy and influence in ventilator decision-making.  Decisions to change ventilator settings, including FiO2 (91%), ventilator rate (65%) and pressure support (57%) made independently by nurses.  Mean number of nurses employed per ICU bed:   - 4.7 in Australia - 4.2 in New Zealand   All units report 1:1 nurse:patient ratio for ventilated patients |
| A comparative study examining the decision-making processes of medical and nursing staff in weaning patients from mechanical ventilation | Taylor F | 2006 | 5 concepts identified:   - Treatment – optimising the patient, returning them to their previous norm, establishing treatment alongside weaning - Balance – physiological margin, work and rest, compensation - Making and maintaining progress – assessing, changing, reassessing; maintaining progress achieved; gradualism - The individual – patient responses, past medical history, working with the patient |
| Evaluation of the Perceived Barriers and Facilitators to Timely Extubation of Critically Ill Adults: An Interprofessional Survey | Balas MC, Tate J, Tan A et al | 2021 | 80% of clinicians believed current SBT and extubation process takes too long and the delay negatively impacts patient outcomes. Most common reasons were:   - SBT timing - Low provider confidence in making extubation decisions - Patient-specific factors   Strategies to overcome barriers include:   - Automated extubation protocol - Performing SBT when physician with responsibility for decision-making present - Reducing clinician perception of reprimand or condemnation for failed extubation |
| Perceived responsibility for mechanical ventilation and weaning decisions in intensive care units in the Kingdom of Saudi Arabia | Alkhathami M, Al-Haddad M & Alenazi A | 2022 | Physician directors and respiratory therapists collaborate on most key decisions, with limited nursing involvement. Physician directors perceived to have greater autonomy and influence in ventilation and weaning decision-making. |
| Understanding interprofessional decision-making processes and perceptions of oxygenation for acute respiratory failure | Curtis B, Rak K, Richardson A et al | 2020 | Themes identified:   - Professional role in oxygenation management - Rationale for adjusting PEEP vs FiO_2_ – all providers favoured titrating FiO_2_ over PEEP due to rapid response and favourable side effect profile - Conceptions of hyperoxaemia – well defined but physiological effects poorly understood - Aspects of conservative oxygenation therapy implementation – rationale understood but several barriers to implementation identified   Physicians were more comfortable with lower SpO_2_ ranges than nurses  Inter-professional decision-making characterised by:   - Nurses focus on patient assessment with subjective and objective criteria and did not manage ventilator parameters - Respiratory therapists follow protocol in implementing goals of care - Physicians value condition-driven treatment |
| Nurses' near-decision-making process of postoperative patients' cardiosurgical weaning and extubation in an Italian environment | Villa G, Manara D & Palese A | 2012 | Pattern of nurse decision-making based on 3 themes:   - Collect data and suggest a decision - Standardise competency - Maintain adherence to unwritten rules   Nurses rarely start weaning or extubate patients independently. Instead:   - Collect data - Consult with other nurses - Recommendation ultimately overseen by physician |

**Supplementary Table 2 – Decision-making when weaning from mechanical ventilation**

| Title | Authors | Year | Factors identified |
| --- | --- | --- | --- |
| Continuous care and patients' basic needs during weaning from mechanical ventilation: A qualitative study | Khalafi A, Elahi N & Ahmadi F | 2016 | Three themes identified:  Time spent with patient   - Continuous communication - Long, face-to-face contact - Time spent building trust   Comprehensive supervision   - Continuous response to fluctuating indices - Holism - Comprehensive objective-subjective view - Experiential perceptiveness   Maintenance of quality of care during shifts   - Effective communication between shifts - Overnight stop - Intelligent management |
| Understanding nurses' decision-making when managing weaning from mechanical ventilation: a study of novice and experienced critical care nurses in Scotland and Greece | Kydonaki K, Huby G, Tocher, J et al | 2016 | Series of initial cues that identify and confirm whether a patient is ”weanable”, spread across 7 criteria and cross 3 phases (pre-weaning, weaning, transition to T piece or extubation):   - Gas exchange* (assessed through FiO_2_, SpO_2_, blood gas values, ventilator settings) - Work of breathing* (ability to take spontaneous breaths, breathing pattern, respiratory rate, tidal volume, RSBI, return of airway reflexes (e.g. cough)). - Level of consciousness (sedation, GCS, level of agitation, pain) - Lung condition* (underlying pathology and its resolution as seen through imaging, secretion burden, strength of cough) - Knowledge of patient (frailty, episodes of desaturation, inability to clear secretions, stable on minimal support) - Cardiovascular stability (amount of inotrope support, presence of arrhythmias, fluid overload) - Signs of infection (prexia, inflammatory markers, amount and character of secretions)   * most likely to be used at the time of extubation  Psychological concepts for describing differences in approach to weaning:   - Focus gambling – likely to change more than 1 ventilator setting at a time - Conservative focusing – likely to change 1 feature at a time |
| Weaning from mechanical ventilation: factors that influence intensive care nurses' decision-making | Tingsvik C, Johansson K & Martensson J | 2015 | Overall theme: complex nursing situation where patient receives attention which is influenced by the current care culture.  Sub-themes:   - Individual overall assessment of the patient leads to consensus - Lack of consensus creates inconsistent care culture   Factors that influence weaning:   - Patient’s physical status – breathing parameters, time on mechanical ventilator, poor physical condition, underlying disease process - Patient’s mental status – level of sedation, cognitive function - Patient’s perspective – patient’s wishes and experiences, the environment - Nurse’s professional knowledge – experience, knowledge in respiratory care, area of responsibility, intuition - Nurse’s personality – professional approach, attitude to respiratory care and weaning - Nurse’s possibility of being present – working situation, ability to observe the patient, continuity of care - Conditions under which team work – guidelines, team members attitude to ventilator management, possibility of making autonomous decision, collaboration, availability of physician, presence of structured weaning plan |
| Weaning from mechanical ventilation: a scoping review of qualitative studies | Rose L, Dainty KN, Jordan J et al | 2014 | Overall themes:   - Interprofessional collaboration and communication important - Healthcare professionals combine subjective knowledge of the patient with objective clinical data - Protocol-driven weaning needs to be balanced against individual patient needs   Nurse’s ability to wean patients dependent on:   - Role and scope of practice - credibility, accountability and autonomy within a team, influence of technology - Information that drives decision-making - objective and subjective information, the role of decision-making tools - Influence on weaning outcome - nurse-patient relationship, the role of the interprofessional team   Physician’s ability to wean patients dependent on:   - Tools or factors to facilitate weaning decisions – protocol or tool-related issues, objective and subjective information, time of weaning, teamwork or team interaction - Perceptions of nurses’ role and scope of practice – nurse competence, experience and interaction with nurses |
| Critical care nurses management of prolonged weaning: an interview study | Cederwall CJ, Plos CK, Rose L et al | 2014 | Critical care nurses drive the weaning process using both a patient centred and targeted approach   1. Individual planning  - Creating an individual plan - Creating continuity - Evaluating the process  1. Assessing patient capacity  - Condition - Respiratory function - Wellbeing - Resources - Reactions  1. Managing the process  - Initiating the process - Prioritising time for weaning - Driving the process forward  1. Managing team interaction  - Collaborative planning - Dialogue with the physician |
| Perceived decisional responsibility for mechanical ventilation and weaning: a Norwegian survey | Haugdahl HS, Storli S, Rose L et al | 2014 | - Work of breathing - Patient well-being - Evidence of clinical deterioration |
| Anaesthetists' perceptions of facilitative weaning strategies from mechanical ventilator in the intensive care unit (ICU): a qualitative interview study | Pettersson S, Melaniuk-Bose M & Edell-Gustafsson U | 2012 | Four categories that govern decision-making:   - Instrumental strategy – optimisation of physiology through nursing interventions, medication and technical tools - Interacting strategy – teamwork, physician’s interaction with the patient and family members, provision of psychological support - Process-orientated strategy – goal-related, individual adjustment - Structural strategy – competence, organisation |
| The factors which influence nurses when weaning patients from mechanical ventilation: findings from a qualitative study | Lavelle C & Dowling M | 2011 | - Physiological influences – patient assessment, oxygenation, ventilator settings, use of diagnostic tests - Clinical reassessment/ decision-making - Nurses’s experience, confidence and education – experience, confidence, education, scope of practice, intuition - Patient’s past medical history and current ventilation – past medical history, the “patient themselves”, psychological factors - Intensive care working environment – medical colleagues, nature of intensive care nursing, scans/ re-sedation - Use of a weaning protocol |
| Accuracy and reliability of extubation decisions by intensivists | Tulaimat A & Mokhlesi B | 2011 | Four most common reasons to postpone extubation:   - Acid-base status interpretation as acute respiratory acidosis - Breathing pattern – as assessed by tidal volume, respiratory rate, RSBI, minute ventilation - Mental status – GCS - Secretions – amount of secretions on tracheal suctioning   Accuracy of predicting who would be successfully extubated was low – sensitivity 57%, specificity 31%, AUC 0.35. |
| The decision-making processes of nurses when extubating patients following cardiac surgery: an ethnographic study | Hancock HC & Easen PR | 2006 | Nurse-led weaning dependent on:   - Unwritten physiological criteria - Nursing practice, which in turn depends on:   - Level of education   - Compliance with unit-based practice   - Attitudes to leadership and responsibility – defined by experience, confidence, willingness to take responsibility and patient complexity   - Availability of resources – access to senior support and technology |
| A study exploring factors which influence the decision to commence nurse-led weaning | Gelsthorpe C & Crocker C | 2004 | Three themes to emerge from the data   1. Decision making  - Professional accountability - Experience - Justification for decision - Protocol vs professional judgement  1. Pathophysiological factors  - Time of day - Change in physical parameters - Improvement in organ function - Co-morbidity - Preparation for weaning  1. Multi-professional team working  - Support |
| Protocolized weaning from mechanical ventilation: ICU physicians' views | Blackwood B, Wilson-Barnett J & Trinder J | 2004 | Primary themes identified:   - Information required for weaning decisions and clinical judgement - Professional boundaries - Protocol issues - Timing of weaning   Three types of information used:   - Empirical objective – resolution of state which required mechanical ventilation e.g. recovery of conscious level; most/ all patient’s physiological variables in the normal range - Empirical subjective – through clinical examination e.g. symmetrical chest movement - Abstract – intuitive component which arises from experience |
| Factors influencing the patient during weaning from mechanical ventilation: A national survey | Mårtensson IE & Fridlund B | 2002 | Factors that guide decision to wean include:   - Nutrition – weight, albumin, glucose - Metabolism – PaCO2, pH, base excess - Measurable parameters – bedside observations, blood gases - Ability to communicate – pencil and paper, lip reading, alphabet board, signs for yes and no - Psychological wellbeing – daily routine, need for information, stress factors, social network - Analgesics and sedatives used |

**Search Strategy (applied to MEDLINE (Ovid) and EMBASE**

1     Decision Making, Shared/

2     Decision Making/

3     ((make or making or made or individual* or informed or share? or sharing) adj2 (decid* or decision* or choice? or choos*)).ti,ab,kf.

4     1 or 2 or 3

5     exp Respiration, Artificial/

6     ((assist* or mechanical) adj2 (respiration or ventilation)).ti,ab,kf.

7     ((positive adj2 (airway pressure or pressure breathing or pressure ventilation or pressure support)) or (cpap or peep or bipap)).ti,ab,kf.

8     (((volume controlled or pressure controlled or intermittent) adj2 ventilation) or (CMV or IMV or VCV or PCV or SIMV)).ti,ab,kf.

9     5 or 6 or 7 or 8

10    implementation science/

11    implement*.ti,ab,kf.

12    decision support techniques/

13    Decision Support Systems, Clinical/

14    (decision? adj3 (aid? or support or system? or process*)).ti,ab,kf.

15    (barrier? or challenge? or obstacle? or enable? or facilitat* or influence* or oppurtunit*).ti,ab,kf.

16    10 or 12 or 13 or 14 or 15

17    4 and 9 and 16
